# Supporting Regulatory Measures in the Context of Big Data Applications for Smart Grids

**DOI:** 10.3389/fdata.2021.675461

**Published:** 2021-09-21

**Authors:** Mihai A. Mladin

**Affiliations:** ^1^ Romanian Energy Center, Bucuresti, Romania; ^2^ Research, Development and Innovation Department, Bucuresti, Romania

**Keywords:** big data and analytics, regulatory measures, supporting "cost of service"—TOTEX approach, ICT chapter requirements and updates within network codes, Smart grids (intelligent networks)

## Introduction

Just mentioning big data in any context, we refer practically to a huge source of information whose processing can ensure the development of algorithms and the substantiation of decisions with minimal or almost no margin of error.

The large volume that practically ensures the critical mass of information represents the power of big data, but the effective exploitation of this valuable source depends, to a large extent, on the quality of the data analysis, interpretation algorithms, and the associated ICT applications.

In more and more fields, big data and in general digitalization is following a trend with a very high dynamics. In business, digitalization most often refers to enabling, improving, and/or transforming business operations and/or business functions by leveraging digital technologies and a broader use of digitized data, turned into intelligence and actionable knowledge.

The energy sector is increasingly associated with the terminology "energy transition," and the main factors that determine and enhance this transition are the integration of renewable energy sources and digitalization. By default, and with technology advancement, the market reacts and proposes solutions to respond to these trends, but their adoption and implementation depends, to a large extent, on the regulatory framework.

## Key Regulatory Measures in Support of Digitalization and Big Data Applications for Smart Grids

The energy sector is certainly the one in which historical data on the operation of energy systems have been collected to a large extend over time. Moreover, the trend and dynamics of digitalization consolidate this positioning of the energy sector toward big data and the exploitation of this huge potential.

With the increase of distributed energy and the challenges of integrating variable renewable energy, the main actors in the field, TSOs and DSOs, are increasingly able to collaborate and take on new roles. The trend of digitalization in supporting smart grid processes by adopting and implementing data-driven solutions also generates new actor profiles such as solution integrators, aggregators, and others who play key roles in this context of energy transition.

### Regulations to Support “Cost of Service”

Intelligent energy systems for monitoring and operating energy networks rely on the adoption and implementation of big data applications, which, at an advanced level, can be outlined in intelligent machine learning solutions. In most "go-to market" scenarios that are analyzed in energy research projects, these solutions are integrated and defined in the business model projections as services.

Going further on these scenarios, the integrators of such solutions, as aggregators or other new profiles outlined in this context, become the providers of these services. TSOs and DSOs, as potential beneficiaries, need appropriate policies and regulatory framework to encourage the solution acquisition technically and financially and generally their adoption on the specific market.

An impactful regulatory measure, meant to become an important lever in the context described above, refers to the provision of incentives and support for "cost of service."

**FIGURE 1:**
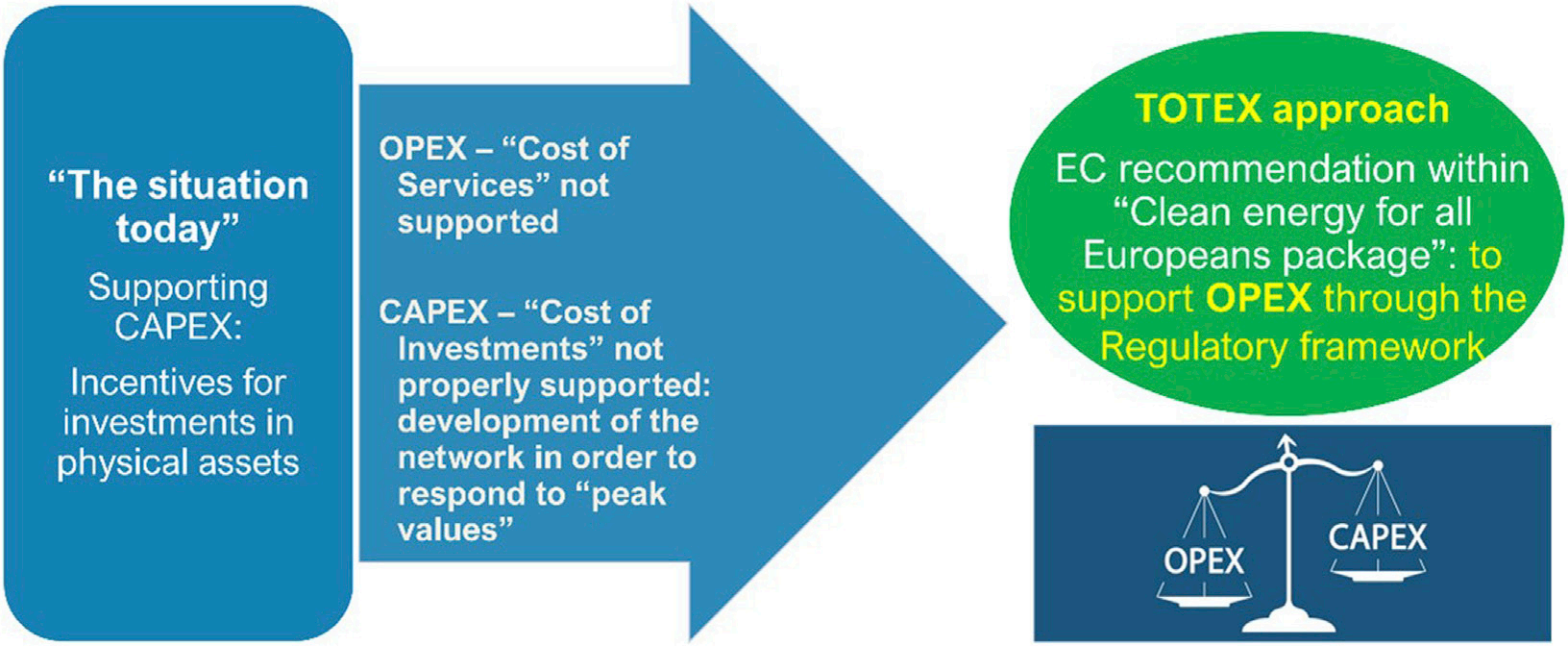
TOTEX approach.

To a large extent, the national energy markets of many European countries already have regulatory provisions to encourage and support investments in the development of energy networks aimed at improving the quality of their operation, which are related to CAPEX. The same objective, of operational safety and increasing the quality of energy services, can be achieved more efficiently through a mix of CAPEX and OPEX solutions, the latter reducing the intensity of the financial effort that should be supported by energy market operators (Figure 1).

In a broader OPEX support framework, the policies to incentivize should consider not only hardware components such as sensor acquisition for better insights regarding electricity grid status but also telecommunication solutions for implementation of faster and more reliable data transmission.

### ICT Chapter Requirements and Updates Within Existing Network Codes

In the same context and from the same perspective of regulation, an important aspect refers to ICT chapter requirements within existing network codes.

At the European level, TSOs have a well-developed and well-founded regulatory framework. Most of the research work has been focused on a time perspective up to 2020 and on the implication of RES at the transmission network level. However, it is widely accepted that much of the growth of renewables beyond 2020 may be based on decentralized generation. So far, no thorough analysis has been conducted beyond the transmission level, which means that the distribution networks are, at the current state, insufficiently analyzed and tested, which may result in additional future challenges through unidentified behavior.

Regarding transmission, the European Commission has defined a set of network codes with two associated objectives: the first leading to the completion of the EU internal energy market and the second to achieve the 20% target for renewable integration by 2020. Therefore, this initial target of 20% renewable energy sources (RESs) was the basis for the definitions of the current set of network codes, and the existing design components of the ancillary services are meeting the same criteria.

**FIGURE 2:**
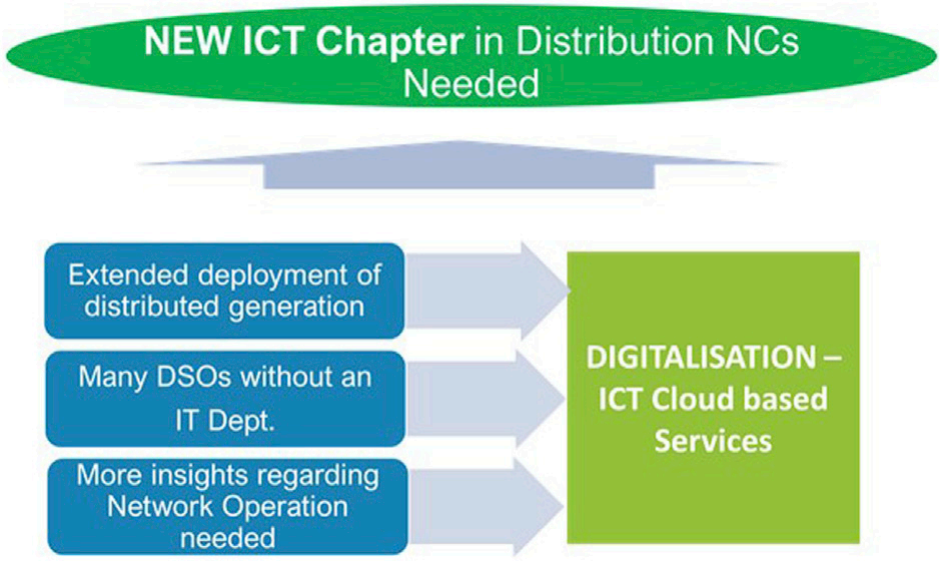
ICT chapter in energy distribution NCs.

On the way to exponentially increase variable renewable energy and digitalization, several technical and regulatory challenges need to be considered and amendments to the existing network codes and ancillary services are necessary. It refers to a series of critical changes and adaptations, from a technical point of view, as frequency and voltage control, to support the stability, safety, and optimal operation of the energy system, with regulatory implications both from this perspective and from that of ICT aspects, as is the case of ICT chapter requirements within existing network codes (Figure 2).

It responds, of course, to the context of exponential increase in the degree of involvement of ICT technologies in energy solutions, to ensure, primarily, compliance with uniformity and transferability requirements and properly supporting the main technical challenges:

- to enable new technics for voltage stability control in the power grid by connecting a huge number of communication end points in the future
- to enable the new technique for online inertia estimation that will solve the current problems, that is, decreased system inertia because of penetration of distributed energy sources
- to enable the new technique for frequency stability control that will solve the existing power grid problems, that is, to reduce the frequency variance

## Conclusions—Source of Well-Founded Regulations

Both regulatory measures described above are critical for the effective support of big data applications and energy digitalization from the DSO and TSO perspective, within the smart grid context.

Regulatory proposals with a direct impact, as well as others that fall within the broad regulatory context and address both technical and market challenges, should be properly linked and respond to a set of key regulatory principles of the governance framework for future electricity networks, as described below.

(i) Efficiency of the investments and costs

It is well known that, in the power sector, all the costs are in the end included in the energy price and, therefore, covered by the end-user. At the same time, maintaining an affordable and sustainable electricity price on long term is a major goal for all EU members. Under these conditions, it is very important to optimize the adopted technical and regulatory measures to achieve the maximum impact for the safety in operation of the power systems, closely monitoring the financial impact as well.

(ii) Collaboration at the regional level

Natural resources are not equally distributed among EU members, and therefore, it is necessary to increase the collaboration beyond the national borders, to make full use of the existing capabilities. The cost for the activities necessary for the day-to-day operation of the power systems must be optimized at a regional level rather than the national level, as is today. Putting in practice of this principle will require, in the first place, the harmonization of the regulatory and legislative framework among EU members and, in the second place, development of regional structures such as control and coordination centers able to provide a proper resource transfer when needed.

(iii) Transparency and predictability

One of the most important results of the unbundling was the development of many companies and firms, private or state owned, linked together in a very intricate activity. In many cases, the economic interest of these legal entities was contradictory or they were in a direct competition for providing services or resources. Obviously, the society’s interest is to support the development of those entities that are helpful for the power system operation and to restrain the development of the entities that are only taking advantage of different administrative of regulatory mismatches, thus increasing, for not good reason, the electricity prices at the end-user’s level. Providing a transparent and predictable regulatory and legal framework will help the existing or potential investors in the power sector to develop business plans sustainable on long term, and as a result, the electricity price will be under control.

(iv) Priority

Taking into consideration the complexity of the power systems and electricity markets’ operation and the challenges generated by the transition from nowadays’ situation to up to 100% RES, it is very important to accurately identify the priority scale of the necessary measures. It is well known that a good rule may have bad results if it issued too early or may have no results if it is issued too late (or something in between), so the timing of the regulations is of outmost importance. A proper identification of the priority and, sometimes, urgency of a measure will bring benefits from both the time point of view (by reducing the overall duration of the process) and financial point of view (by effectively supporting the next steps and, thus, reducing the costs of the whole process).

(v) Long-term continuity

The energy transition process built on higher renewable energy integration and digitalization needs to be designed and followed as a whole. It is necessary to make sure that measures taken in the first stages, although apparently useful at that moment, are not hindering the implementation of future stages by becoming obstacles that must be removed. In this way of thinking, every step must be coordinated with the existing and future conditions and necessities so that both are answered properly.

(vi) Societal acceptance and involvement

Acceptance and involvement of the society must not be approached separately because they are synergically connected. Acceptance will bring more involvement and more involvement will bring more acceptance, supporting, in this way, the development process towards digitalization and implicitly big data applications. Based on the experience gained so far in implementation of several projects on digitalization of energy, the abovementioned principles are not independent of each other, and they are connected in a hierarchical structure. The most important principle proved to be “societal acceptance and involvement.” Failing to follow this principle will, most likely, lead to significant difficulties in implementing the necessary measures, no matter how much they are justified from the technical and economical point of view. On the contrary, the action plans developed according to this principle proved to be much more easy and even cheaper to be put in practice.

The principles described above were the basis for the analysis and the algorithm for defining and substantiating the proposals for completing the regulatory framework. These have been the subject of extensive consultations with all categories of stakeholders, especially in the implementation of the RESERVE Project, 2020,^1^ and the SOGNO Project, 2020^2^. They have been validated together with energy experts and policy makers, and more than that, they are on the agenda of the relevant organizations belonging to the European Commission on the path to their adoption.

From the perspective of the same principles and on the same basis of analysis, the activity of collecting arguments and substantiating these proposals continues not only within the EdgeFLEX Project, 2020,^3^ but also in other projects with which the members of the consortium strive to act synergistically.

## Footnotes

1 RESERVE Project - Renewables in a Stable Electric Grid (2020). Bucharest: EU. (website: Available at: https://www.re-serve.eu).

2 SOGNO Project – Service Oriented Grid for the Network of the Future (2020). Bucharest: EU (website: Available at: https://www.sogno-energy.eu).

3 EdgeFLEX ProjectProviding Flexibility to the Grid by Enabling VPPs to Offer Fast Dynamics Control Services (2020). Bucharest: EU(website: Available at: https://www.edgeflex-h2020.eu).

## Abbreviations

CAPEX, Capital Expenditure; DSOs, Distribution System Operators; ICT, Information and Communications Technology; OPEX, Operating Expense; RES, Renewable Energy Source; and TSOs, Transmission System Operators.

